# An Investigation into Mechanical Properties of 3D Printed Thermoplastic-Thermoset Mixed-Matrix Composites: Synergistic Effects of Thermoplastic Skeletal Lattice Geometries and Thermoset Properties

**DOI:** 10.3390/ma17174426

**Published:** 2024-09-09

**Authors:** Saleh Khanjar, Srimanta Barui, Kunal Kate, Kameswara Pavan Kumar Ajjarapu

**Affiliations:** Mechanical Engineering Department, Shumaker Research Building, University of Louisville, Kentucky, KY 40208, USA; saleh.khanjar@louisville.edu (S.K.); srimanta.barui@louisville.edu (S.B.);

**Keywords:** mixed matrix composite, fused filament fabrication, thermoplastic-thermoset, mechanical properties, fractography

## Abstract

This study aims to develop thermoplastic (TP) and thermoset (TS) based mixed matrix composite using design dependent physical compatibility. Using thermoplastic-based (PLA) skeletal lattices with diverse patterns (gyroid and grid) and different infill densities (10% and 20%) followed by infiltration of two different thermoset resin systems (epoxy and polyurethane-based) using a customized FDM 3D printer equipped with a resin dispensing unit, the optimised design and TP-TS material combination was established for best mechanical performance. Under uniaxial tensile stress, the failure modes of TP gyroid structures with polyurethane-based composites included ‘fiber pull-out’, interfacial debonding and fiber breakage, while epoxy based mixed matrix composites with all design variants demonstrated brittle failure. Higher elongation (higher area under curve) was observed in 20% infilled gyroid patterned composite with polyurethane matrix indicating the capability of operation in mechanical shock absorption application. Electron microscopy-based fractography analysis revealed that thermoset matrix properties governed the fracture modes for the thermoplastic phase. This work focused on the strategic optimisation of both toughness and stiffness of mixed matrix composite components for rapid fabrication of construction materials.

## 1. Introduction

Thermoplastics (TP) are widely known for their ductility, higher elongation, toughness and easy processability, but at the cost of ultimate tensile strength (UTS). On the other hand, thermoset (TS) materials are generally stiffer, brittle, higher mechanical properties (strength and modulus) and difficult to process once cured [1,2,3]. To import these twin unique qualities in a single material system, it has been a several decades-long efforts of many researchers to make them compatibilized [4,5,6,7]. In brief, thermoplastic and thermoset composites are of a major research interest for their unique and tailorable properties for structural applications, such as automobile body manufacturing, aerospace interior applications as well as sports gears [8,9,10,11,12,13]. While improvement of mechanical properties of thermoplastics and thermosets by reinforcing stiffer materials, e.g., carbon fibers, glass-ceramic fibers and particulates as well as metallic fibers and particles are practiced, on the reverse side, incorporation of thermoplastic materials in brittle thermosets to improve the inherent toughness and ductility are also reported [14,15,16]. Since the last two decades, additive manufacturing (AM) dominated the arena of ‘fast fabrication with infinite complexity’ in a sophisticated and accelerated manner, where mould-based manufacturing lacks the efficiency in terms of cost, time, and complexity where on-demand design-specific manufacturing is considered [16,17,18,19,20,21]. While it is too challenging to process thermosets using AM techniques as they demonstrate time dependent curing, thermoplastics are instead fabricated in a design-specific manner followed by infiltration/impregnation/dispensing with thermoset materials. Kong et al. developed a two-matrix continuous fiber-based composites using short fiber reinforced polyamide (SFRPA), polyamide (PA), polylactic acid (PLA) and epoxy resin. Using a co-extruding printer assembly, they printed fibers pre-impregnated with thermoset fused with thermoplastics to form a dual matrix composite. Additional thermoplastic material was co-printed as a binder between the shell and the core filaments [20]. In a recent work, Chauvette et al. developed a non-planar structure of thermoplastic (high temperature reinforced polymer, HTRP) using FFF technology integrated with a 6-axis robot arm. After fabricating the curved TP based sandwich panel, the curvature was scanned following generation of G-Code toolpath for the thermoset dispensing. An epoxy and amine hardener based thermoset resin system was mixed with hollow glass microspheres and dispensed on the curved surfaces with the help of 6-axis robotic arm multi-nozzle dispensing system to create micro-scaffolds as the abradable layers [22]. 3D printed (FFF) PLA-based skeleton reinforced rigid polyurethane foam (RPUF) based composite was developed by Tao et al., who demonstrated that lattice structures and density played significant roles on compression and flexural performance, further endorsed by FEA based simulation studies. A global enhancement of the mechanical properties was attained through a mutualism relationship, where the skeletal lattices strengthened the RPUF, while the RPUF helped in achieving the stability of the skeletal struts [23]. In another study, using a co-curing process, thermoplastic films were sandwiched between thermoset pre-pregs and it was evidenced that plasma-based surface modification of the thermoplastic enhanced the TP-TS bonding evaluated using lap shear strength assessment. It was shown that, additional roughening of the thermoplastic surface further enhanced the bonding strength [24]. In many engineering applications, thermoset composites show low toughness and brittle behavior whereas, thermoplastic composites demonstrate limited rigidity although having commendable toughness. To harvest the unique properties of thermoplastics and thermosets in a single material system, Goodarz et al. developed a hybrid composite system comprising glass fiber reinforced thermoplastic (GTP) and glass fiber reinforced thermoset (GTS). As a positive effect of interlayer hybridization using GTP, the failure strain of GTS improved significantly, in turn improving the toughness of the hybrid system [25]. Taken together, it is worthwhile to mention that both thermoplastic and thermoset have their unique properties, which after an intelligent amalgamation, can achieve better physical properties for structural and load bearing applications. While extensive endeavor has been placed to make the TP and TS compatible using surface modification and chemical treatments, the effects of physical interlocking of these two incompatible phases using different geometric patterns are insufficiently appreciated in literature.

In this study, we emphasize on the fabrication and characterization of various TP-TS mixed matrix composite, exploring the influence of various infill patterns and densities on the mechanical properties. Deploying advanced FFF 3D printing technology and utilizing two distinct infill designs: gyroid and grid structures, along with different infill densities, this research aimed to investigate the synergistic effects of combining different materials and infill configurations on the overall tensile strength of the final specimens. The manufacturing process involved the FFF-based 3D printing of PLA as the thermoplastic porous skeletal lattices with different geometries, followed by infiltration with two diverse thermoset systems using a customised resin dispensing assembly: a polyurethane (flexible) and an epoxy-based (stiffer) resin. The comprehensive analysis contributes to a deeper understanding of the interplay between infill design, density, and different resin systems with varying flexibility, offering valuable knowledge for the optimization of mixed matrix composite structures from off-the-shelf materials. The design dependent physical interlocking mechanism between the TP and TS phase is the major contributory knowledge disseminating in this research, without any additional interfacial compatibility, e.g., inter-phase chemical bonding, interplay with surface roughness, surface wettability modification with plasma treatment etc.

## 2. Materials and Methods

### 2.1. Design of Mixed Matrices with Different Design Elements and Infill Density

**Figure 1:**
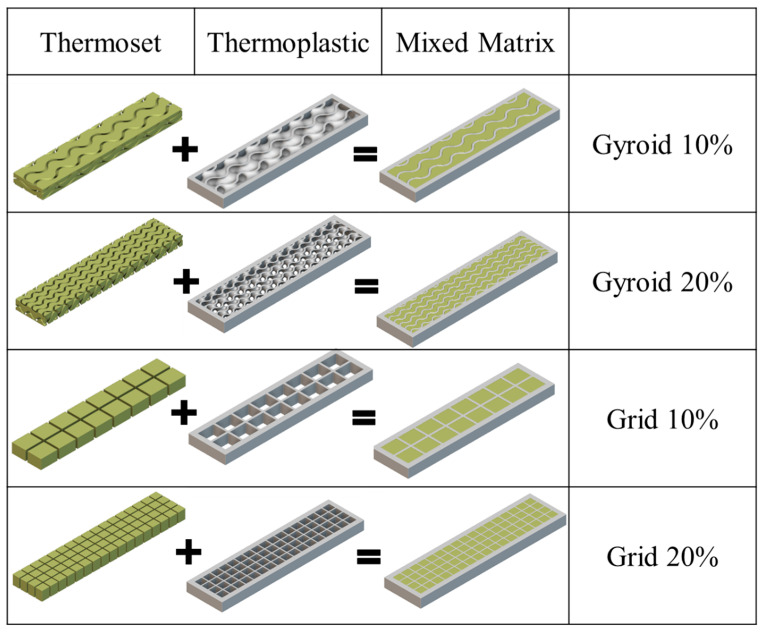
nTopology designs showcasing the thermoset phase (**left**), PLA thermoplastic phase (**middle**), and the resulting hybrid composite structure for Gyroid and Grid designs at 10% and 20% infill densities (**right**).

Computer aided porous designs were generated with two distinct infill patterns: the grid and the gyroid, to quantitatively probe the influence of the interlaid thermoplastic designs on the mechanical properties. Two infill densities of 10% and 20% were also considered to investigate the effect of the interfacial area between the thermoplastic and thermoset on the strength properties. The infill lattices were generated using Cura (Ultimaker, Utrecht, The Netherlands) Slicer and Gcode generator with defined lattice parameters. The 10% and 20% infilled Gyroid walled lattice from the TPMS family were constituted by the unit cell size of (12 × 12 × 5) mm^3^ and (6 × 6 × 5) mm^3^ respecticvely. Whereas, the Grid infill variants with 10% and 20% infill density comprised of the unit cell dimnsions of (9.5 × 8 × 5) mm^3^ and (4.5 × 4 × 5) mm^3^ respecively. The surface area of the 10% and 20% infilled designs of both variants (grid and gyroid) were quantified using nTopology™ (3.41.2) to rationalise the effect of interfacial interractions between the PLA (thermoplastic) interlaid structures with the infiltrated and cured thermoset (epoxy and polyurethane). Figure 1 represents the mixed matrix design variants along with the interactive thermoplastic and thermoset components. We also measured the weight of 3D printed parts (thermoplastic skeleton) and the final weight of all the mixed matrix geometries after infiltrating different thermosets, to comparatively study the amount of thermoset uptaken by the thermoplastic skeleton with different infill densities and their influence on the mechanical properties.

Rectangular tensile test coupons specimens having the overall dimension of (80 × 20 × 5) mm^3^ were developed using the above-described infill patterns and densities. The traditional “dog-bone” structure of tensile test specimens could not be adapted in this study to address a couple of challenges underlying the design properties. To design the “dog-bone”, one of the following two approaches need to be adapted. First, narrowing down the unit cell dimensions around the gauge which will make it more condensed giving rise to asymmetrically higher strength in that region. In the second approach, keeping the unit cell dimensions unaltered in the gauge region which can eliminate few of the crucial components of the design lattice which will significantly compromise in the tensile strength. Keeping these twin catches in consideration, to record the true tensile properties of the structures, it was necessary to keep the cross-section homogeneous throughout the length of the tensile specimens.

### 2.2. 3D Printing of PLA Thermoplastic Skeleton and Silicon Based Thermoset Casting Molds

A fused filament fabrication (FFF) 3D printer (Anycubic Kobra, Shenzhen, China) equipped with direct drive extrusion was used to create rectangular test coupons. The 3D printing process parameters to print PLA were as follows: Nozzle Diameter—0.5 mm, Print Speed—50 mm/s, Print Temperature—200 °C, and Layer Height—0.25 mm. The infill density, which varied between 10% and 20%, coupled with infill type distinctions (Gyroid, Grid), was a critical consideration. The top and bottom solid layers were intentionally omitted to expose the infill, which would later facilitate thermoset casting processes.

**Figure 2:**
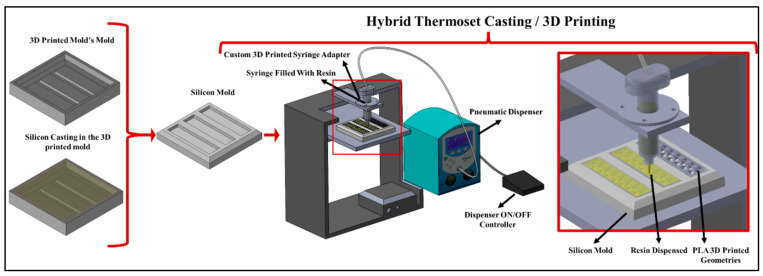
Custom mold fabrication process involving 3D printing of customised CAD, silicon casting within the 3D printed mold, and the resulting flexible silicon mold. On the right, the PLA skeletons were kept in the silicon mould and the custom designed resin infiltration assembly dispensed the epoxy and polyurethane resin ‘on-demand’.

To create a hybrid composite structure, a custom mold was designed and 3D printed using silicon as the base material. The mold was tailored explicitly for the mixed matrix geometry, providing a precise structure for subsequent casting. A FDM 3D printer (MakerGear M2, Beachwood, OH, USA) was modified by removing the filament extrusion head and replaced with a 3D printed syringe adapter. The resin filled syringe was attached to a pneumatic source (Nordson Ultimus II, Westlake, OH, USA) to infiltrate the PLA skeletal lattices ‘on-demand’ (Figure 2).

### 2.3. Thermoset Infiltration to Fabricate Mixed Matrix Composites

**Figure 3:**
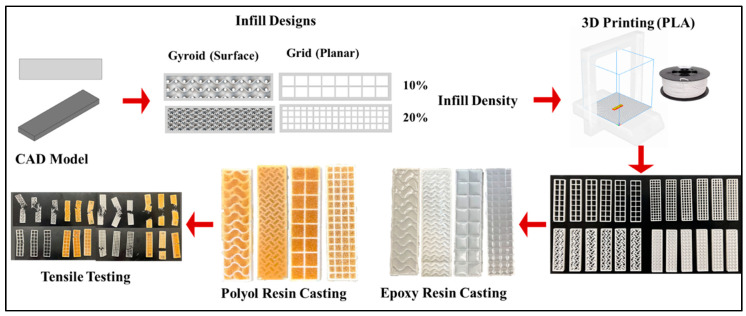
Schematic representation of the process of developing hybrid mixed matrix composites (thermoset-thermoplastic).

Commercially available polyurethane and epoxy based resins were used to infiltrate the 3D printed PLA skeletons. The polyurethane Resin System consisted of two parts—Part A, SETATHANE^®^ D 1150, and Part B, DESMODUR VL—mixed at a ratio of 70% Part A and 30% Part B. The epoxy resin system also comprised two parts—Part A and Part B Terroxy resin—mixed at a ratio of 80% Part A and 20% Part B. polyurethane and epoxy resins were strategically selected to have a flexible and stiff matrix properties, respectively, intended to achieve different responses under mechanical loading conditions. A centrifuge-based mixer and simultaneous degassing system was used to mix the two-part thermoset resins with a mixing speed of 2000 rpm for 30 s. Next, the 3D-printed PLA parts with different geometries and infill densities was inserted into the previously fabricated rectangular silicon molds and the thermoset resins were dispensed using the modified cartesian 3D printer equipped with a fluid dispensing unit and left to set overnight. Care was taken to ensure uniform and controlled distribution of the thermoset material within the hybrid composite structure. Further, a waiting period of one week at room temperature was observed before to ensure complete curing before proceeding to mechanical tests and chemical analysis. The overall process is schematicaally represented in Figure 3.

### 2.4. Chemical, Topographic and Physical Characterizations

To comprehensively assess different physico-chemical properties of the hybrid composite, a set of qualitative and quantitative characterizations were performed. To qualitatively analyse the molecular structure of the hybrid mixed matrix composite and to probe the curing quality, Fourier Transformed Infrared (FTIR) spectroscopy (Agilent Cary 630 Santa Clara, CA, USA) was used in the wavenumber range of 500–4000 cm^−1^ (Attenuated total reflectance mode). Mechanical properties of the hybrid composite were evaluated quantitatively using an Universal testing Machine (UTM, Instron 5569A, Norwood, MA, USA). Tensile tests were conducted on the specimens using 50 kN load cell at a cross head speed (CHS) of 25 mm/min, providing valuable insights into the material’s strength and deformation behavior. Optical microscopy (Keyence VHX-S750E, Osaka, Japan) was employed to analyze the fractured surfaces of the specimens and investigate the interfacial adhesion between the thermoplastic and thermoset resin. To analyze the fracture surfaces in a comprehensive manner, field emmision gun based scanning electron microscopy (FEG-SEM, Apreo 2, Thermofischer Scientific, Walthan, MA, USA) was used for both epoxy and polyurethane based mixed matrix composites. Weight of each test coupon was measured post-casting and curing, to build a quantitative understanding of the resin infiltration and density within the hybrid matrices. The comprehensive characterizations, combining mechanical, chemical, and microscopic analyses, offer critical insights about the hybrid composite’s properties and performance.

## 3. Results

### 3.1. 3D Printing of PLA Patterns and Manufacturing of Mixed Matrix Composites

The optimised printing parameters reproducibly fabricated the near-net-shaped grid and gyroid patterened PLA skeletons. In line with the initial hypothesis, the total surface area of the gyroid infill design, as calculated using nTopology surface area measurement tool, showed 63% higher value compared to the grid infill design with 10% density. Although having higher surface area in the gyroid structure, the PLA skeleton weight for both gyroid and grid structures were same which was 2 g. The grid infill design with 20% density had a surface area of 7379 mm², while the gyroid infill design with 20% density measured 8821 mm², representing a 20% increase in surface area. In this case also, the PLA skeleton weight remained the same, i.e., 3 g, for both gyroid and grid structures.

These designs were infused with two different thermoset systems, polyurethane resin and epoxy resin. For the polyurethane resin, the weight of the grid infill designs with 10% and 20% density were 8.2 g and 8.3 g, respectively. For the polyurethane-infused gyroid designs, the weight of the 10% and 20% infill densities was 7.5 g and 7.65 g, respectively. For the epoxy resin, the weight of the grid infill designs with 10% and 20% density were 10.7 g and 11 g, respectively. For the epoxy-infused gyroid designs, the weight of the 10% and 20% infill densities were 10.7 g and 10.8 g, respectively. Table 1 provides details of the surface area, weight, and thermoset infill characteristics of different infill designs and densities were extensively compared for 3D printed mixed matrix parts in this study.

### 3.2. Fourier Transformed Infrared Spectroscopy Analysis

**Figure 4:**
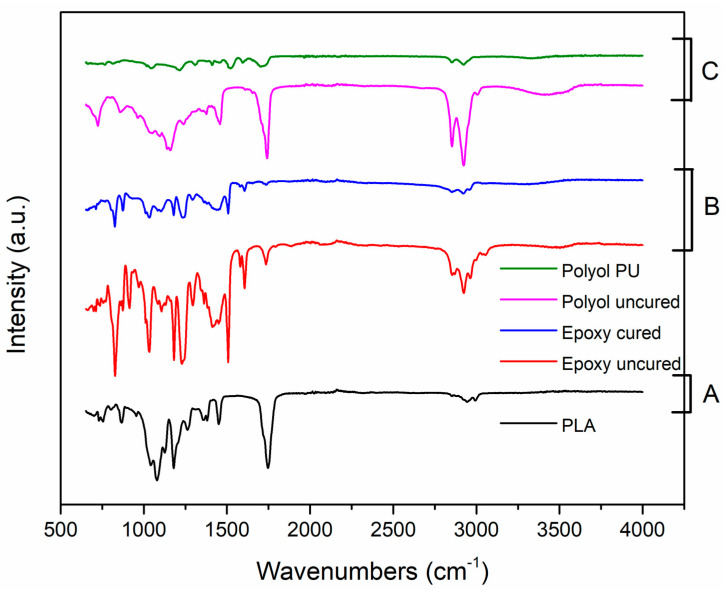
FTIR spectroscopy revealing the chemical structure of the skeleton PLA (**A**) and different thermoset matrix resins [epoxy (**B**) and polyurethane (**C**), both cured and uncured].

Figure 4A represents the FTIR spectrum of the skeleton PLA. The wavenumbers of 1077 cm^−1^ and 1181 cm^−1^ represent C-O stretching, whereas the later one is the most characteristic absorption of ester bond. The peaks at 1368 cm^−1^ and 1450 cm^−1^ originate from the C-H bending whereas a strong band at 1750 cm^−1^ is the characteristic of C=O stretching vibration. The twin peaks at 2996 cm^−1^ and 2946 cm^−1^ are assigned to C-H stretching of -CH_3_ groups of PLA [26,27,28].

The peak locations at the FTIR spectrum were similar in both uncured and cured epoxy-based resin having no detectable amine peaks (Figure 4B). This implicated that the hardener reacted completely with the epoxy monomer during the curing process. The significant reduction in the intensities of the characteristic peaks indicated the effective curing of the epoxy in presence of amine hardener. The IR bands at 824 cm^−1^ and 913 cm^−1^ represented the stretching of (C-O-C) and (C-O) bonds of the oxirane group of epoxies, respectively. The peaks at 1031 cm^−1^ and 1242 cm^−1^ denoted stretching of ethers (C-O-C) and asymmetrical aromatic C-O stretch. While the peaks at 1505 cm^−1^ and 1609 cm^−1^ represented stretching vibrations of C-C and C=C bonds of aromatic rings, asymmetrical C-H stretch of -CH_2_ group as well as C-H stretch of C-H_2_ and C-H bonds related to the aromatic and aliphatic groups were observed at 2922 cm^−1^ and 2965 cm^−1^ respectively [29,30,31].

For the polyurethane PU resin-based matrix, the chemical signatures of virgin polyol and curing of the same in the presence of diisocyanate were probed qualitatively [32,33,34] (Figure 4C). Wavenumbers of 955 cm^−1^ and 1090 cm^−1^ represented unsaturation (C=C) and ether linkage C-O-C of the virgin polyol. While bending vibration of C-H bonds in the –CH_2_ groups was observed at 1460 cm^−1^, a strong absorption peak assigned to the C=O stretching of ester was found around 1735 cm^−1^. The peaks at 2860 cm^−1^ and 2925 cm^−1^ denoted symmetrical and Asymmetrical stretching of CH_3_ and CH_2_ respectively. A broad band around 3450 cm^−1^ originated from the structural -OH groups.

After curing, the characteristic functional groups of polyurethane (PU) were recorded at the wavenumbers of 1600 cm^−1^, 1700 cm^−1^ and 3300 cm^−1^ for carbamate bond (C-N), carbonyl urethane group (-C=O) and amide urethane bond (N-H) formation. There was no characteristic peak for -NCO group present in the PU, this stands for the fact that during curing, all diisocyanate group reacted with the polyols (major phase) to form the polyurethane matrix [35].

### 3.3. Mechanical Properties

**Figure 5:**
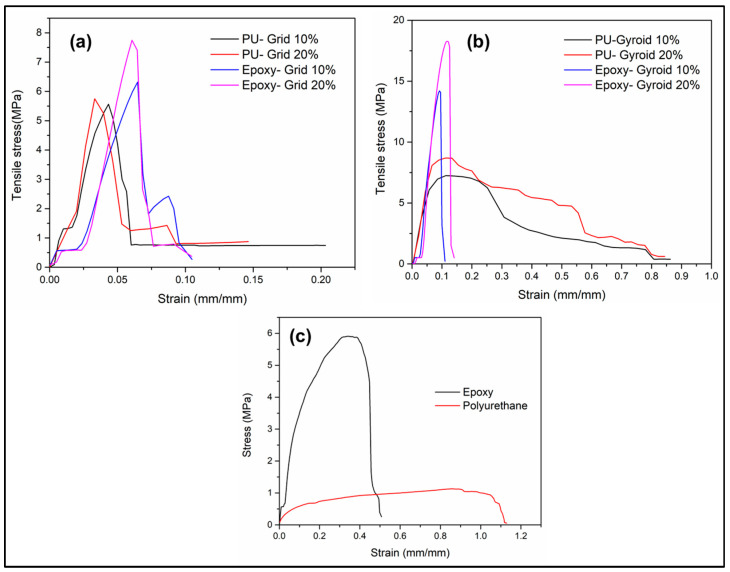
Representative stress-strain curves for mixed matrix composites of epoxy with PLA and polyurethane with PLA for (**a**) Grid and (**b**) Gyroid infill designs. The uniaxial tensile behaviour of the baseline materials are shown in (**c**).

At least three specimens were examined for each design under tensile stress for statistically meaningful results. Figure 5a,b illustrated representative stress-strain responses for Grid and Gyroid epoxy-based and polyurethane-based mixed matrix composites, respectively. Figure 5a shows the stress-strain curve for Grids specimens, while Figure 5b shows the same curves for specimens with Gyroid infill patterns.

**Figure 6:**
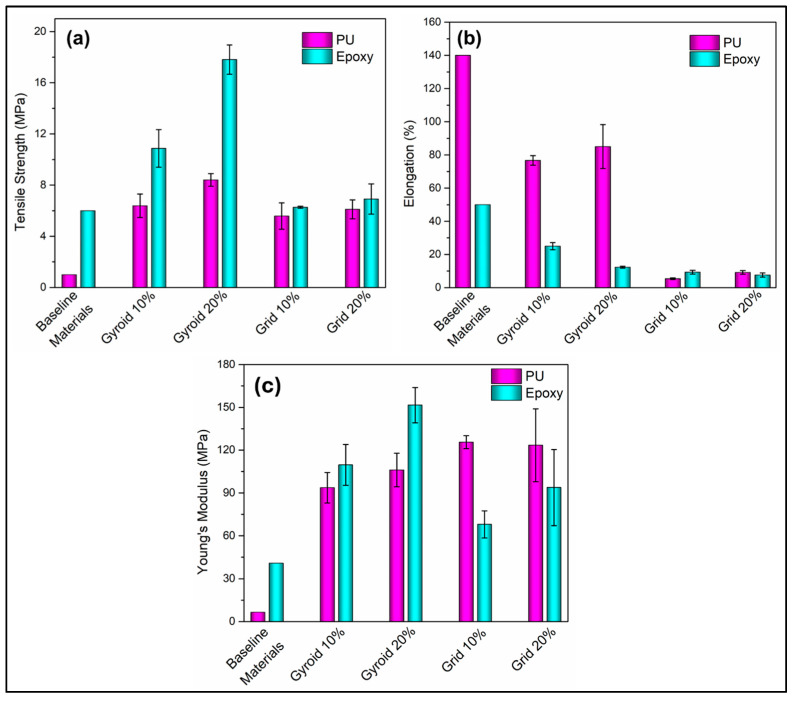
Summary of the ultimate tensile strength (UTS) and % elongation of 3D printed PLA thermoplastic—epoxy and PU based thermoset mixed matrix composites. (**a**) demonstrates the UTS for the grid and gyroid infill designs with two infill densities for both types of resin infiltrations, while (**b**) showcase(ed the % elongation values of the same specimens. Young’s moduli of all the baseline and mixed matrix composites are represented in (**c**).

All the tensile strengths were observed in the range of 5–10 MPa, except for Gyroids geometries infilled with epoxy resin, which had a higher tensile strength range of 14–18 MPa. All strains were in the range of 0.5–0.1, except for gyroids infilled with polyurethane resin, which had a higher strain of around 0.8. Figure 6a,b provided mean and standard deviations for ultimate tensile strength (UTS) and % elongation. Notably, the strength characteristics of different designs and infill densities were scrutinized. For Gyroid infill with 20% density, the ultimate stress was 17.8 MPa with epoxy and 8.4 MPa with polyurethane. Gyroid with 10% density exhibited 10.9 MPa and 6.4 MPa maximum stress for epoxy and polyurethane, respectively. Grid infill, at 10% density, showed ultimate tensile strengths of 6.3 MPa with epoxy and 5.6 MPa with polyurethane, while at 20% density, the values were 6.9 MPa and 6.1 MPa, respectively.

Interestingly, the effect of infill density showcased a couple of intriguing trends. For instance, changing the Gyroid mixed matrix infill density from 10% to 20% with polyurethane increased strength by 30% and elongation by 10%. In comparison, the same change with epoxy resulted in a 63% strength increment and 100% reduction in elongation. Grid mixed matrix also exhibited varying responses to different infill densities. Overall, Gyroid designs consistently demonstrated higher ultimate stresses compared to grid designs, and transitioning from polyurethane to epoxy thermoset systems substantially enhanced the mixed matrix’s stress resistance, exemplified by a 260% increase in tensile strength for the 20% Gyroid infill with epoxy compared to the 10% Grid infill with polyurethane. These findings provide valuable insights into the nuanced interactions between infill design, density, and thermoset systems, informing the optimization of mixed matrix structures in structural engineering applications.

### 3.4. Qualitative Assessment of the Fractured Surfaces

**Figure 7:**
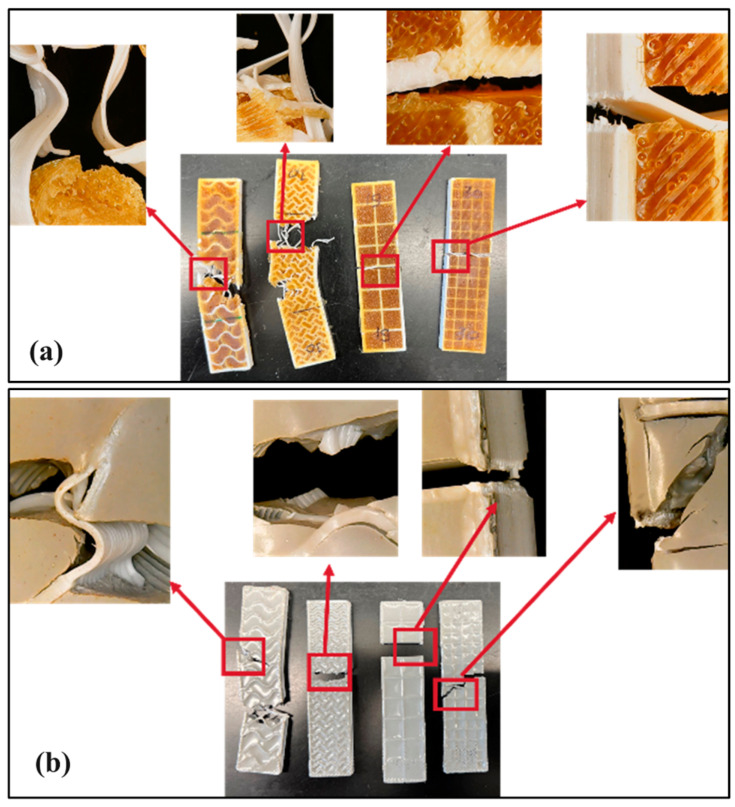
Optical microscope images of the fractured surfaces after tensile tests. The top panel (**a**) shows the fractured interfaces of different mixed matrices with PLA–polyurethane system, while the bottom panel (**b**) represents the fracture behavior of the specimens with PLA–epoxy based mixed matrix composites after tensile tests.

Optical image analysis on the failed regions after tensile tests revealed a set of crucial knowledge about the interfacial interactions in the mixed matrix composited of thermoplastic and thermoset (Figure 7a,b). As there is no interfacial chemical bonding, the stability of the interface largely depends on the physical adhesion between the two phases. As expected, composite with gyroid structured thermoplastic skeleton displayed ‘delayed failure’ comprising a significant ‘fiber pull-out’. This is a typical characteristic of fiber reinforced composites demonstrating the better reliability under structural applications. Whereas, in case of grid like structure, the failure was catastrophic, as all the thermoplastic cells were isolated, and the thermoset resin was also confined inside the cells. This allowed no ‘crosstalk’ with the neighboring cells and failure of one cell dictated the failure for the entire structure. Being interconnected in nature, both the thermoplastic skeleton and the resin matrix in the gyroid structure could effectively distribute the stress throughout the length in the direction of the axial tensile stress, contributing positively to resisting the force against failure, which was absent in the grid structures. Interestingly, being physically flexible compared to epoxy, the composites having polyurethane resin showed an extent of ductile deformation (Figure 7a), whereas the failure of epoxy resins was clearly brittle in nature, like a glassy fracture (Figure 7b). The failure characteristics were found to be consistent in both infill densities of 10% and 20% for a corresponding design feature (grid and gyroid).

### 3.5. Fractography Study with SEM Image Analysis

**Figure 8:**
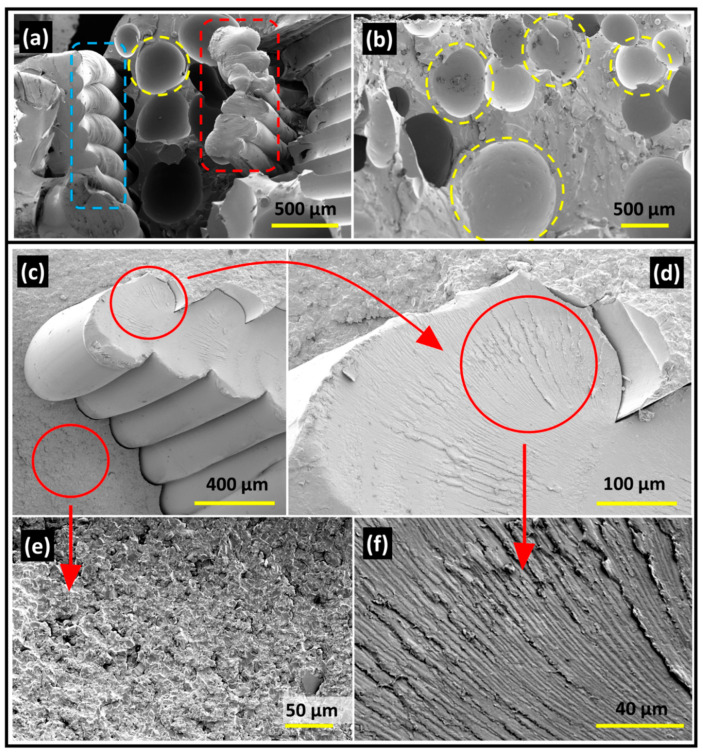
Scanning electron microscopy-based fractography studies for PLA skeletal lattice-based composites of epoxy and polyurethane matrices. Top panel (**a**,**b**) demonstrates the failure trend of PLA-polyurethane based mixed matrix composites, while the bottom panel (**c**–**f**) represents the brittle failure modes of the PLA-epoxy based composite systems.

Figure 8 shows the fractured surfaces of PLA-polyurethane and PLA-epoxy based mixed matrix composites where distinctly different modes of fracture are observed. Figure 8a,b represents the fracture surfaces of PLA-polyurethane based composites. ‘Cup and Cone’ type ductile mode of fracture was observed in the PLA phase (red dotted rectangle) of the PLA-polyurethane composite, where the matrix (polyurethane) also demonstrated the presence of many ‘cups’ showing similar ductile failure. The yellow dotted circles highlight the formation of the cups in the polyurethane matrix. The ‘necking’ was also observed in the 3D printed PLA skeletal fibers (blue dotted rectangle).

A completely different trend of fracture was observed in the PLA composites of epoxy-based matrices. Figure 8c–f represents the brittle nature of failure for the PLA-epoxy based composite system. The epoxy matrix represented brittle fracture which was shown in Figure 8c and further magnified in Figure 8e. In Figure 8c, the V-shaped ‘chevron’ ridges were found which pointed back towards the center of the origin of the failure in the respective PLA filaments which was magnified in Figure 8d, and further resolved in Figure 8f (radiating fan-like pattern) for the best comprehension. It was important to mention that the fracture behaviors were identical irrespective of the infill densities of PLA, indicating the dominating influence of the matrix material in dictating the fracture behavior.

## 4. Discussion

The Holy Grail of this study is to learn how different thermoplastic-based infill designs with different infill densities affect the mechanical properties of mixed matrix composite, infiltrated with both stiff and flexible thermoset resins. In particular, we looked at the impact of two infill designs–a grid design and a gyroid design, on the thermoset and thermoplastic based composite strength properties. The grid design was chosen for its non-continuous pathways, creating discrete regions for thermoset infiltration. By examining the absence of continuous pathways, we could determine how it influences the distribution and interaction of thermosets within the composite. On the other hand, the gyroid design was picked for its continuous pathways that facilitate interconnected infiltration of the thermoset phase. By incorporating a design with continuous pathways, we aimed to investigate the impact on the overall homogeneity and strength of the composite structure. To further analyze the effect of surface area on composite properties, we selected two infill density percentages—10% and 20%. As we know, increment in the infill density has a linear relationship with the total surface area, this allowed us to examine how different infill densities influenced the physical properties by altering the extents of interfacial interactions between the thermoplastic and thermoset phases. This returned us the logical correlation between the structure and performance of the composite matrices.

### 4.1. Influences of Infill Patterns and Infill Density on the UTS and % Elongation

Gyroid infill designs consistently outperformed their grid counterparts when UTS is taken under consideration. This appears in line with the expectation that the Gyroid structure, with its interconnected pathways, provides more homogeneous stress distribution along the direction of the force application, resulting in delayed failure with higher strength values than the grid patterned structures. While gyroid structured composites exhibit higher strength properties, while compared between the 10% and 20% infill density of the same design variants, the strength increased significantly with the infill density. This observation was consistent for both polyurethane and epoxy based composite systems. Higher infill density in the gyroid structure provided closer interaction between the PLA thermoplastic and the continuous resin system giving rise to the robust interfacial adhesion of the parallel and interconnected phases. This is believed to be the reason for the enhanced UTS in the 20% infill gyroid patterned composites.

Interestingly, grid infill designs exhibited mixed responses to different infill densities. It has been recorded that there was no significant difference between the 10% and 20% infilled grid designed composites, although mean values of the 20% infilled structures were found to be slightly higher in both polyurethane and epoxy matrices. This typical behavior may have been arisen from the restrained response of the individual PLA cells under tensile strength. Because of the isolated cellular structures of the PLA phases individually containing resin system could not take part in the effective distribution of stress throughout the structure. As a result, failure in any local cell resulted in catastrophic failure in the global structure. With increment in the infill density was not sufficient to overcome this general characteristic for the grid patterned composite, resulting similar UTS in both 10% and 20% infilled designs.

While the resin system is kept unaltered, there is a consensus of decreased elongation as infill density increases. This is particularly evident in Gyroid designs with epoxy resin, where elongation decreases by 100% when density shifts from 10% to 20%. This can be explained for the fact that high density of the PLA skeleton patterns provides more hindrance in deformation. In addition, higher surface area with the higher infill density contributed to higher physical adherence between the associated dissimilar phases, resisting the deformation under uniaxial tensile stress.

### 4.2. Impact of the Various Resin Systems on the UTS and % Elongation

In this study, judicious choice of two different resin variants (stiff and flexible) returned a plethora of interesting results, which are very important in mixed matrix composite fabrication for structural applications. From the Figure 5 and Figure 6, it is evident that across various infill designs and densities, the UTS is consistently higher when using the epoxy resin system than the polyurethane-based resin. Shifting from polyurethane to epoxy thermoset systems consistently increases the UTS in the mixed matrix, as exemplified by the 260% increase in the 20% Gyroid infill with epoxy compared to the 10% Grid infill with polyurethane-based system. Interestingly, this behavior was at the expense of the % elongation, where the % elongation was always higher in case of polyurethane-based resin composites (except the grid design 10% infill). This was expected as polyurethane-based resin is more flexible when compared to its epoxy-based counterpart.

Combining the UTS and % elongation together, in the gyroid structured mixed matrix composites (Figure 5b), for both the 10% and 20% infill density, polyurethane-based composites demonstrated a very high elongation resulting in much higher ‘area under curve’ compared to the epoxy systems. This indicates the potential of the Gyroid patterned composites with polyurethane resin matrix, in the structural applications where high toughness is preferred over higher strength, for example as a high shock absorbing material system.

### 4.3. Influence of Thermoset Matrix on the Failure Behaviour of the Skeletal Lattice Thermoplastic

In line with the observation under the optical microscope, ductile and brittle nature of fracture were found in the cases of PLA-polyurethane and PLA-epoxy based mixed matrix composites, respectively, through SEM based fractography analysis. Higher extent of ‘matrix-PLA skeletal debondingʼ was observed in the cases of PLA-polyurethane based composite where it was less severe in the case of PLA-epoxy composite. The ductile ‘cup and cone‘ failure mode was associated with this system while brittle fracture was found in the PLA-epoxy system. This phenomena could be justified from the perspective of effective energy transfer between the thermoset matrix and the thermoplastic fibers under tensile stress. Being ductile in nature, under tensile stress, the PLA fibers got debonded from the polyurethane matrix resulting in delayed energy transfer to the PLA fibers. This resulted in slow and ductile mode of failure for the PLA phase. On the other hand, in contrast, the brittle and catastrophic crack propagation in case of epoxy matrix, transferred a massive quantum of energy in the PLA fibers all of a sudden, providing no opportunity for necking and matrix debonding, causing simultaneous brittle failure in the PLA fibers. This provides a unique and very important understanding on how different matrix material properties change the failure modes of the same thermoplastic material.

Returning to the initial hypothesis that the choice of thermoplastic skeleton patterns and the strategic selection of thermoset material significantly influence the strength properties of mixed matrix composites. Understanding these trends is crucial for optimizing the design and fabrication of such composites for light weight structural engineering applications. Based on the engineering design and 3D printing technology, this study invested the efforts to compatibilize off-the-shelf thermoplastic and thermoset materials, without any additional interfacial chemical bonding to contribute to low-cost structural fabrication. For example, building materials as well as shock absorbing storage or base material for engineering instruments. One of the few major limitations of this study was the need to carry the thermoset 3d printing in a separate stage after 3D printing the thermoplastic filament. This is because the thermoset resin has mild expansion behavior during curing, requiring higher curing time. Additionally, the need for post processing after the hybrid 3D printing process, such as polishing and surface preparation. The fabrication approach can further be scaled-up for 3D printing of larger structures using thermploastic filaments for the skeleton then infilled with variety of thermoset resins for rapid 3D printing of larger constructions, for example temporary shelter in various strategic sectors. Deployable industrial scale conveyer belt-based 3D printers are there, which can rapidly fabricate infinite length of life-like structures.

## 5. Conclusions

This study reports a novel approach in physical compatibilizing of thermoplastic and thermoset materials using a design-based perspective. The key outcomes of this research are summarized henceforth:

1. Total eight variants of different TP-TS specimens comprising two different PLA-based skeletal lattice geometries (grid and gyroid), two varying infill densities (10% and 20%) of the same and two different resin systems (epoxy and polyurethane) were developed to figure out the best design and material combination for physically compatible mixed matrix composite.
2. For a given infill pattern, higher infill density gave rise to higher surface area reflecting in the increased intake of resin (weight)
3. Using qualitative FTIR spectroscopy, the signature peaks of the TP and the TS are identified whereas missing and/or decreased intensities of the peaks denoted the complete or partial curing for both the two-part resin systems.
4. For both gyroid geometries, epoxy based mixed matrix composites demonstrated higher UTS compared to their polyurethane counterpart. This was explained in view of the higher stiffness of epoxy compared to relatively flexible polyurethane matrix. Grid geometries showed a mixed behavior.
5. Higher elongation was recorded in the cases of gyroid patterned PLA skeleton-based composites with polyurethane resin system. The elongation increased with the infill density.
6. This behavior was explained in the light of relatively flexible nature of the polyurethane matrix compared to the epoxy system, added with the gyroid geometry which being fully interconnected in the direction of stress application, distributed the load more efficiently prior to failure.
7. From the optical microscope image analysis, ductile nature of failure was identified in the gyroid based geometries where typical “fiber pull-out” was noticed. All the grid-based composites (for both epoxy and polyurethane-based matrices) showcased brittle and sharp fracture. As the TS resins were confined in the isolated cells of the grid structure, there was no provision of the stress distribution like the continuous and interconnected gyroid structure. As a result, failure of any one cell dictated catastrophic failure of the entire structure, where more “progressive failure” was observed in the gyroid counterparts.
8. Having higher area under curve for the 20% infilled gyroid patterns infiltrated with polyurethane resin, this composite system was believed to be the material with highest toughness. These mixed matrix composites can be used in high mechanical shock absorbing applications.
9. Fractography revealed that thermoset matrix material properties (ductile or brittle) governed the failure modes of the thermoplastic lattice skeletons. While the PLA fibers demonstrated ductile failure when embedded in polyurethane matrix, brittle fracture was observed in the same PLA fibers in the PLA-epoxy system.

## Figures and Tables

**Table 1:** Comprehensive Comparison of 3D Printed Mixed Matrix Parts, Highlighting Surface Area, Weight, and Thermoset Infill Characteristics for Different Infill Designs and Densities.

Infill Design	Grid		Gyroid	
Infill Density %	10	20	10	20
Design Geometry	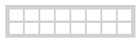	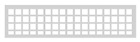	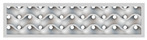	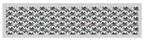
Weight, g (Thermoplastic)	2	3	2	3
Surface Area, mm^2^	4665.5	7379.5	7640.8	8821.2
Weight, g (with Polyurethane Resin)	8.2	8.35	7.5	7.65
Mixed Matrix (with Polyurethane Resin)	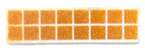	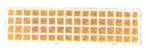	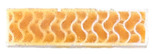	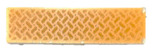
Weight, g (with Epoxy Resin)	10.7	11	10.7	10.8
Mixed Matrix (with Epoxy Resin)	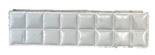	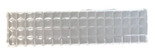	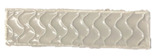	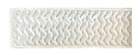

## Data Availability

Materials are available upon request.
